# Rerouting of Host Lipids by Bacteria: Are You CERTain You Need a Vesicle?

**DOI:** 10.1371/journal.ppat.1002208

**Published:** 2011-09-01

**Authors:** Agathe Subtil

**Affiliations:** 1 Institut Pasteur, Unité de Biologie des Interactions Cellulaires, Paris, France; 2 CNRS URA 2582, Paris, France; Duke University, United States of America

Fifteen years ago, in a series of elegant studies, Hackstadt and colleagues showed that the obligate intracellular bacteria *Chlamydia trachomatis* save on their lipid needs by incorporating sphingomyelins (SMs) made by their host [1]–[3]. Shortly after, Hatch and McClarty's teams reported that several eukaryotic glycerophospholipids are also trafficked from the host to the bacteria, which replace host-synthesized straight-chain fatty acids by their own branched-chain fatty acids [4]. Even cholesterol, a lipid rarely found in bacteria, was shown to accumulate in *Chlamydia* [5]. As a result of this intense exploitation of host lipids, the composition of the bacterial membrane is closer to that of a eukaryotic cell than to that of a prokaryote.

Throughout their developmental cycle, chlamydiae reside within a membrane-bounded compartment, the inclusion. How they acquire host lipids remains an open question. Possible mechanisms studied so far involve vesicular trafficking from host compartments, including vesicular traffic out of the Golgi apparatus, fusion with multivesicular body–derived vesicles, and engulfment of lipid droplets [6]. Two papers recently published in *PLoS Pathogens* show that non-vesicular traffic is also involved [7], [8].

**Figure 1:**
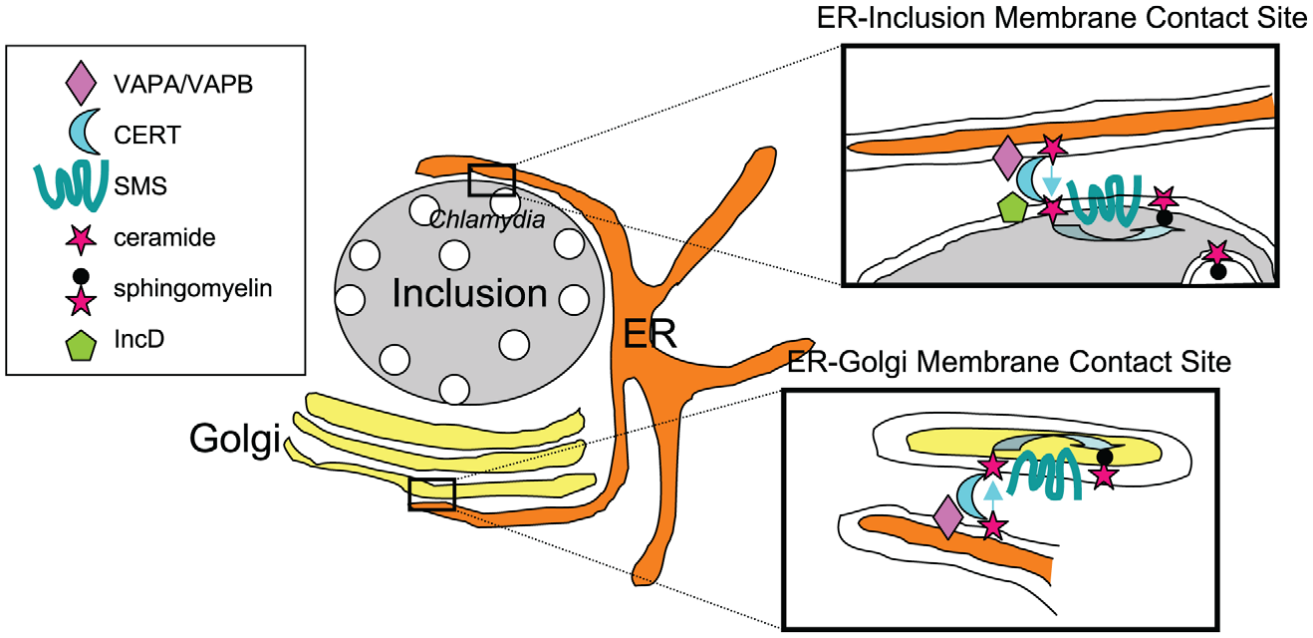
Direct transport of ceramide from the ER to *C. trachomatis* inclusion. At ER-Golgi membrane contact sites the ceramide transfer protein CERT associates to the ER-resident proteins VAPA/VAPB and, via its PH domain, to PI4P at the *trans*-Golgi. Upon transfer by CERT, ceramide is converted to SM by a SM synthase (SMS). In *Chlamydia*-infected cells, ER-inclusion membrane contact sites involving VAPA/VAPB and CERT are observed. CERT interacts with the inclusion-anchored bacterial protein IncD through its PH domain but independently of PI4P. Upon transfer to the inclusion membrane, ceramide might be converted to SM by host SMS, which is enriched around the inclusion, and incorporated by the bacteria. Because the catalytic site of SMS is in the lumenal site of the Golgi apparatus, it would imply that the enzyme traffics to the inclusion membrane to convert ceramide, by a mechanism that remains to be determined. Other possibilities for SM acquisition by the bacteria are discussed [8]. Alternative routes for the transfer of SM and other lipids to the *Chlamydia* are discussed in an excellent recent review [6].

SMs are synthesized by the transfer of phosphorylcholine to a ceramide in a reaction catalyzed by SM synthases. When added to infected cells, the fluorescent probe C6-NBD-ceramide traffics through the Golgi apparatus and rapidly accumulates in the bacteria, in the form of SM and not ceramide [1], indicating that the probe is converted to SM by host SM synthases before transport to the bacteria. However, understanding SM acquisition by the bacteria requires going one step back, into ceramide transport. Both studies show that CERT, a lipid transfer protein involved in non-vesicular endoplasmic reticulum (ER) to Golgi transport of ceramide [9], and VAPA and VAPB, its ER-resident partners, are enriched around the inclusion membrane [7], [8]. At the ultrastructural level, Derré and colleagues observed CERT on the inclusion membrane and VAPB on ER tubules in close proximity to the bacteria-filled compartment. By analogy with the ER-Golgi membrane contact sites described for non-vesicular transport of ceramide by CERT (Figure 1), Derré proposes that ER-inclusion membrane contact sites allow for direct transfer of ceramide to the inclusion. The group identified the inclusion protein of bacterial origin IncD as a specific binding partner for CERT [7].

For what purpose does ceramide traffic to the inclusion? Bacteria accumulate an estimated 50% of SM synthesized from exogenously added ceramide [1]. Therefore, while a role for ceramide per se on the inclusion is not excluded, it is expected that its conversion to SM should strongly benefit the bacteria. There are SM synthase genes in humans identified as SMS1 and SMS2. SMS1 is found in the *trans*-Golgi apparatus while SMS2 is predominantly associated with the plasma membrane. Elwell and colleagues show that both enzymes are in close proximity to the inclusion membrane, and propose that the recruitment of CERT, its ER binding partner VAPA, and SM synthases establish an “on-site SM factory” [8].

Like CERT, other lipid transfer/binding proteins have been described as functional components of ER-Golgi membrane contact sites. Future studies need to address whether these non-vesicular lipid transfer systems are involved in the acquisition of phospholipids and sterols by the inclusion. Such a direct transfer could explain why transfer of host phospholipids to the bacteria was unaffected by brefeldin A, which inhibits Arf1-dependent vesicular transit through the Golgi apparatus. It is also consistent with the observation that traffic of glycoproteins out of the Golgi is not disrupted by infection [3].

In the presence of brefeldin A, SM acquisition by the bacteria is reduced and inclusions are smaller [1]. This observation and others argue for the existence of a vesicular-mediated access of SM to the inclusion [2]. The new data presented in *PLoS Pathogens* do not speak against this possibility, which can operate alongside non-vesicular traffic. In fact, Elwell et al. also provide data showing that depletion of the brefeldin A target GBF1 reproduces the effect of the drug on *Chlamydia* infection, implicating GBF1 in the vesicular route for SM acquisition [8].

Interestingly, while brefeldin A (or GBF1 depletion) only affect inclusion size, and not bacterial proliferation, CERT (or VAP) depletion have an impact on both [7], [8]. Does this mean that the non-vesicular process makes a greater contribution to total SM acquisition? This will be difficult to assess with the methods used currently. Due to rapid photobleaching, quantification of the accumulation of fluorescent probes by imaging is technically challenging. Incidentally, the two studies report divergent results on the effect of CERT depletion on SM accumulation in the inclusion assessed by this technique. In addition to not being quantitative with the probes currently available, imaging does not give information on the possible modifications of the fluorescent-tagged lipid in the host or in the bacteria [4]. But more than quantity, the site of SM acquisition at the inclusion might determine its fate. Elwell's data suggest that the two pathways contribute to different aspects of the developmental cycle of *Chlamydia*, CERT being important for bacterial replication and the vesicular pathway being essential for inclusion growth and stability [8]. This would imply that the SMs of different origin constitute two distinct pools, either because they consist of different molecules and/or because they do not diffuse freely on the inclusion and cannot be equally taken up by the bacteria.

Both studies were conducted on the human pathogen *Chlamydia trachomatis*. Surprisingly, Derré and colleagues report that the guinea pig strain *Chlamydia caviae* does not recruit CERT to its inclusion, consistent with the absence of IncD in this strain [7]. Is that so unexpected? We already know that these obligate intracellular bacteria have adopted multiple redundant mechanisms to enter cells and to intercept host intracellular traffic, to give only two well-studied examples [6]. It is hard to imagine that chlamydiae have not put the same energy into exploiting all possible steps of lipid transport in eukaryotic cells.
